# Continuous kidney replacement therapy in critically ill children: heparin vs citrate

**DOI:** 10.1038/s41390-024-03238-9

**Published:** 2024-05-13

**Authors:** Patricio E. Ray

**Affiliations:** Department of Pediatrics and Child Health Research Center, University of Virginia School of Medicine, Charlottesville, VA USA

Continuous kidney replacement therapy (CKRT) is currently the most frequent approach used in pediatric intensive care units (PICU) to treat children with acute kidney injury (AKI), fluid overload, electrolytes-acid base disorders, multiorgan dysfunction (MOD), inborn errors of metabolism, acute liver failure, sepsis, toxic ingestions, and drugs removal.^1^ Preventing the deposition of fibrin and clots in the filters during CKRT in children is a significant challenge. These events progressively reduce the surface available for diffusion and convection, decrease the clearance of solutes, and lead to filter clotting, interrupting therapies and causing blood losses that require blood transfusions. Very few large pediatric studies have been done comparing how the most common anticoagulants used in CKRT affect the clinical outcome of critically ill children. In the current issue of *Pediatric Research*, Atis et al. reported their experience treating 131 critically ill children on CKRT with systemic heparin or regional citrate anticoagulation (RCA) in a tertiary PICU located in Istanbul.^2^ Here, we will review the advantages and disadvantages of the two anticoagulant strategies and discuss key points relevant to the study of Atis et al.

Systemic unfractionated heparin (UFH) was the first-choice anticoagulant used in the 90 s for CKRT, given the experience available using heparin in hemodialysis, its low cost and efficacy to prevent thrombotic events, and rapid reversal with protamine.^1^ Heparin works mainly by enhancing the activity of antithrombin III (AT) inactivating thrombin and factor Xa. Thrombin plays a key role in the coagulation system by converting fibrinogen to fibrin, forming stable clots, activating platelets and coagulation cofactors, and inducing vasoconstriction. All these events are critical to prevent hemorrhages. Therefore, heparin increases the risk of hemorrhages and can also cause heparin-induced thrombocytopenia (HIT), which is induced by antibodies that target complexes of platelet factor 4 (PF4) and heparin and is manifested by thrombotic events.^3^ The diagnosis of HIT, detected in ~1–3% of heparin-treated children,^3^ can be masked by other causes of thrombocytopenia and thrombosis, including the activation and destruction of platelets in the extracorporeal circuit, the peripheral consumption of platelets during sepsis and MOD, and other illnesses that induce AKI and thrombocytopenia (e. g. HUS, TTP). In addition, heparin binds to circulating heparin binding growth factors that induce angiogenesis and vascular leakage (FGF-2, VEGF-A, TNF-$\alpha$), and children with inflammatory/angiogenic diseases are at higher risk of bleeding when treated with heparin.^4^ It is worth mentioning that at Kings College Hospital in London, children <10 kg were treated successfully with prostacyclin (PGI2) during CKRT.^5^ Prostacyclin is a vasodilator that inhibits platelet aggregation and also reduces the amount of heparin needed for effective anticoagulation by inhibiting the local release PF4, which binds heparin neutralizing its activity.

Citrate is an anticoagulant that is injected in the extracorporeal circuit avoiding the systemic anticoagulant effects of heparin. It is both a weak acid and a buffer, and its buffer capacity depends on the proportion of strong cations present in its solution, usually trisodium citrate.^6^ The strong ion difference (SID) of trisodium citrate is zero, and its accumulation in humans can induce mild metabolic acidosis. Since the ionized calcium normally increases in acidosis, a low ionized calcium with acidosis suggest that citrate metabolism is impaired. Sodium-citrate is infused before the filter, acting as an anticoagulant by chelating ionized calcium. The calcium-citrate complexes (CCC) are then removed by filtration and dialysis, and calcium is replaced via an infusion post-filter in the blood returning to the patient. However, CCC can escape into the circulation, where citrate is metabolized to bicarbonate releasing sodium, which further increases the SID and may cause metabolic alkalosis and hypernatremia.^6^ Although citrate accumulation is the most frequent complication of RCA, its most feared side effect is hypocalcemia, which can induce hypotension, convulsions, arrhythmias, respiratory muscle weakness and death. Citrate also chelates magnesium, which should be monitored. In patients with liver failure or poor tissue perfusion, citrate is not metabolized well and may cause a high anion gap metabolic acidosis. Since citrate is not toxic per se, these changes are due to the primary illness that induce the malfunction of the Krebs cycle affecting the metabolism of citrate and pyruvate and leading to the generation of lactate. Citrate associated toxicity is suspected by the presence of low ionized calcium, a total/ionized calcium ratio >2.5, a high anion gap metabolic acidosis, or whenever more calcium is needed to maintain the desired blood calcium levels.^6^ However, recent studies suggested that the Total/ionized calcium ratio is not a reliable indicator to either confirm or rule out citrate accumulation, since changes in albumin, phosphate, lactate, and circulating unmeasured anions can affect this ratio.^7^ Although RCA is avoided in children with liver failure, some pediatric studies showed that after proper adjustments and close monitoring it could be used safely in children with liver injury.

Atis et al. found that RCA is a safe and effective anticoagulation method for CKRT and that prolongs hemofilter survival decreasing the number of red blood cell transfusions after filter clotting.^2^ Their results did not show significant differences in severe bleeding or mortality between groups. In contrast, a large study done years ago by Brophy et al. in 138 children found no significant differences in circuit-filter survivals between those treated with UFH or RCA, but reported a higher incidence of life-threatening hemorrhages in children treated with UFH.^8^ There are several issues that need to be taken into consideration to interpret the findings of Atis et al.^2^ Firstly, although the authors stated that the clinical characteristic of the patients in both groups were largely similar (with the exception of more children with liver failure and hyperammonemia in the heparin group and more tumor lysis syndrome in the RCA group), there were many other clinical differences that despite not being statistical significant when considered individually, appear clinically relevant when considered together. The heparin group included more children who were young, weighted less, required smaller vascular access, suffered more bleeding after catheter insertions, had more catheters inserted in the femoral vein, used lower pump flow rates, and had more MOD. All these factors acting in a simultaneous manner can contribute to filter clotting independently of the anticoagulant used. On the other hand, the RCA group included more children with AKI, fluid overload, catheters inserted in the internal jugular vein (the recommended site), higher BUN levels, lower platelet counts, and more risk factors for bleeding. The PICU mortality was higher in the RCA group (34.5%) compared to the heparin group (23.7%), although this difference was not statistically significant. These data suggest that both groups were not so similar, and may explain why the bleeding outcomes were not significantly different in disagreement with other pediatric and adult studies. Secondly, Atis et al. acknowledged that during the first years of the study all children were treated with heparin, while in subsequent years they were treated with RCA. Thus, the group treated with RCA might have benefited from the CKRT experience acquired during the first years, particularly considering that on average the filters that clotted in children treated with heparin did so rapidly (15 h) when compared to those treated with RCA (38 h) or heparin in other large pediatric studies.^8^ Thirdly, they excluded children with hepatic failure in the RCA group and also children who received anticoagulation therapy for therapeutic purposes during CKRT.

The ultimate goal of CKRT is to improve the clinical outcome and reduce the mortality of critically ill children. Unfortunately, Atis et al. reported that children treated with RCA showed a higher mortality trend.^2^ It is possible that the longer filter clotting times may not necessarily reflect the efficacy or safety of the CKRT procedure, since the filter ability to clear small molecules is impaired before the circuit is clotted, and new strategies can be implemented to monitor the clearance of solutes. Furthermore, a sub-analysis of the RICH trial **(**Regional Citrate Anticoagulation vs Systemic Heparin Anticoagulation), involving 596 adults in Germany,^9^ showed that RCA resulted in longer filter life spans but also significant more new infections compared to heparin.^10^ Since the mortality of children on CRRT continues to be high, it is worth asking why RCA is not associated with lower mortality trends considering its other benefits. The RICH trial was underpowered to assess mortality differences,^9^ suggesting that a very large prospective pediatric study focused on similar illnesses will be needed to answer this question. Also defining how hypocalcemia together with other electrolytes-acid-base disorders that enhance its adverse may affect the mortality rate, might be a difficult task pursuing retrospective chart reviews in which patients with missing values are excluded. Finally, it is worth mentioning that the safety of CKRT-RCA depends on the medical skills, personal experience and technology available in each PICU.^1^ Overall, the final conclusions of Atis et al. are in agreement with most previous adult and pediatric studies and the current Kidney Disease Improving Global Outcomes guidelines. However, further research is needed to improve the outcome of children on CKRT and to define the ideal anticoagulant strategy for each particular clinical case and child.
